# Barrier Properties of Layered-Silicate Reinforced Ethylenepropylenediene Monomer/Chloroprene Rubber Nanorubbers

**DOI:** 10.3390/nano8050314

**Published:** 2018-05-09

**Authors:** Chang Mou Wu, Wen Yen Hsieh, Kuo Bin Cheng, Chiu-Chun Lai, Kuei Chi Lee

**Affiliations:** 1 Department of Materials Science and Engineering, National Taiwan University of Science and Technology, Taipei 10607, Taiwan; cmwu@mail.ntust.edu.tw; 2 Department of Fiber and Composite Materials, Feng-Chia University, Taichung 40724, Taiwan; kbcheng@fcu.edu.tw; 3 Department of Textile Engineering, Chinese Culture University, Taipei 11114, Taiwan; lqj2@ulive.pccu.edu.tw; 4 Taiwan Textile Research Institute, Tucheng Dist., New Taipei 23674, Taiwan; lee@staff.pccu.edu.tw

**Keywords:** layered silicate, nanocomposites, nitroglycerine migration, barrier property

## Abstract

The triacetin and nitroglycerin barrier properties of layered-silicate reinforced ethylenepropylenediene monomer/chloroprene rubber (EPDM/CR) nanorubbers were investigated as rocket-propellant inhibitors. EPDM/CR nanorubbers with intercalated structures were formulated and prepared by the melt-compounding method. The triacetin permeability and nitroglycerin absorption were observed to decrease with increasing layered-silicate content. The layered silicates also improved the flame retardancies of the nanorubbers by forming silicate reinforced carbonaceous chars. Layered-silicate reinforced EPDM/CR nanorubbers are potentially effective rocket propellant-inhibiting materials.

## 1. Introduction

The propelling powers of missiles and rockets are mainly derived from propellants, insulators and shells and the propellants used are flammable and mainly composed of nitroglycerine (NG). Rubber, which has generally been used to inhibit rocket propellants, is formed from macromolecules with loosely packed structures. The function of a rubbery insulator is to dissipate reaction heat through melting and pyrolysis in order to reduce the damage caused by hot gases on the shell of the engine. Because NG molecules are small, they can easily seep into the structures of rubbers. NG migration from the rocket propellant to the inhibitor not only breaks the propellant-insulator bond during storage but also leads to problems such as undesirable ballistics, coning of the burning propellant and degradation of mechanical properties, which may affect the flight trajectory and even cause the insulator to fail (i.e., lose its heat-insulating properties). Therefore, it is necessary for an insulating material to be impermeable to NG, to have sufficient heat-insulating properties and high ablation resistance [1,2,3,4,5].

Ethylenepropylenediene monomer (EPDM) rubbers are commonly used as rubbery insulating materials for solid rocket engines [6,7,8,9,10]. EPDM rubbers are low-density synthetic polymers that have excellent mechanical properties and ablation resistances to a number of chemicals [11,12,13,14]. In addition, they exhibit excellent resistances to ozone, oxygen, heat and weathering degradation; however, they do not completely retard the migration of NG. Chloroprene rubbers (CRs), on the other hand, are heat-, ozone- and oil-resistant. In addition, they have superior ablation and anti-migration properties to some chemicals such as NG [15]. The introduction of electron acceptor groups to the inhibitor, such as chloro groups in rubber molecules, considerably reduces the extent of NG migration from propellants to inhibitors. Therefore, polymer blends of EPDM and CR may enhance the heat and chemical resistance of the rubbery insulator in a solid rocket engine [16,17].

Layered-silicate polymer nanocomposites have been reported to have superior barrier and mechanical properties [18,19,20]. The unique layered organosilicate structure is essential for the manufacture of brand-new high-performance polymer/clay nanocomposites [19]. Compared with conventional filled polymers, layered silicates can improve the mechanical properties [20], gas barrier properties, solvent resistances, heat resistances [21] and flame-retarding capacities of the polymer matrix [22]. Surprisingly, little information is available on the chemical resistances of layered-silicate polymer nanocomposites.

Several methods have been developed to prepare layered-silicate polymer nanocomposites, including in-situ polymerization, polymer intercalation from solution and direct polymer melt intercalation or latex blending. Among these methods, melt intercalation is the most suitable processes for industrialization. Several studies have shown that composites of layered silicates and nanoelastomers, such as EPDM, isoprene rubber (IR), epoxidized natural rubber, silicone rubber and thermoplastic olefins, exhibit excellent mechanical and heat properties [23,24,25]. However, to the best of our knowledge, few studies have been reported that address properties that are important to rocket propellants, namely heat insulation, high ablation resistance and NG permeability. In addition, it has been reported that aramid fibers, in combination with powder fillers such as silica, can be successfully substituted for asbestos in rubbery rocket-motor case insulation without any erosion-resistance loss [26].

Hence, in this work, we studied the NG migration and flame-retardancy behavior of EPDM/CR nanorubbers with different contents of layered-silicate.

## 2. Results and Discussion

### 2.1. Morphology

**Figure 1:**
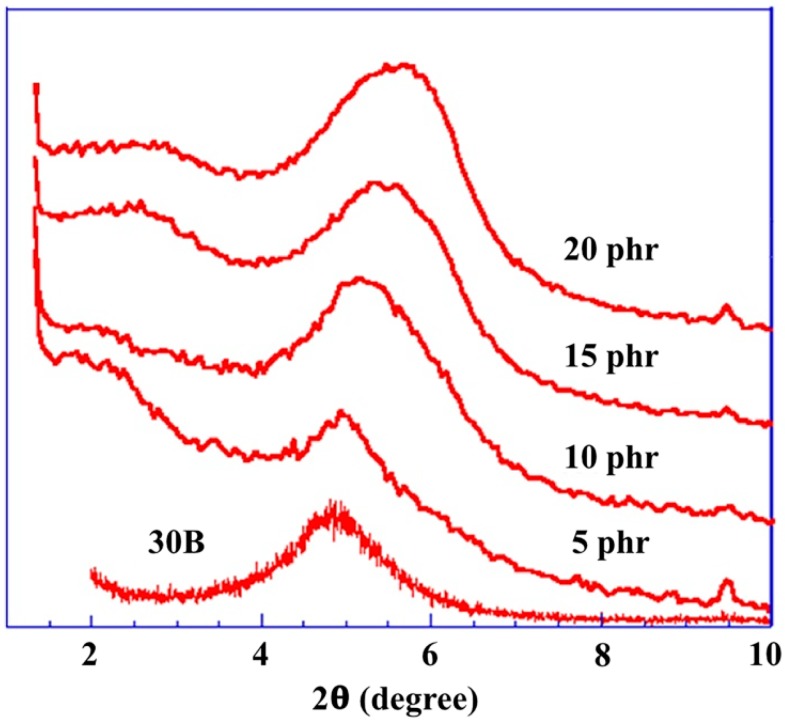
X-ray diffraction patterns of ethylenepropylenediene monomer/chloroprene rubber (EPDM/CR) nanorubbers with various layered-silicate contents.

The efficiencies of the layered silicates in the reinforcing nanorubbers were determined by their dispersions in the matrix and the extent to which the polymer molecules were intercalated between the layered silicates. Figure 1 shows XRD patterns of representative nanorubbers containing 0 to 20 parts per hundreds of rubbers (phr) of layered silicates. The (001) silicate diffraction peaks were observed at lower diffraction angles (2°–2.5°) in the nanorubbers compared to the pure organosilicate (5°). This observation indicates that, through the use of a suitable mixing process and at a certain silicate loading, the viscosity of the nanorubber and the associated shearing force is sufficient to separate the intercalated silicate layers, thereby facilitating the diffusion of the rubber molecules into the intergalleries of the organosilicate.

**Figure 2:**
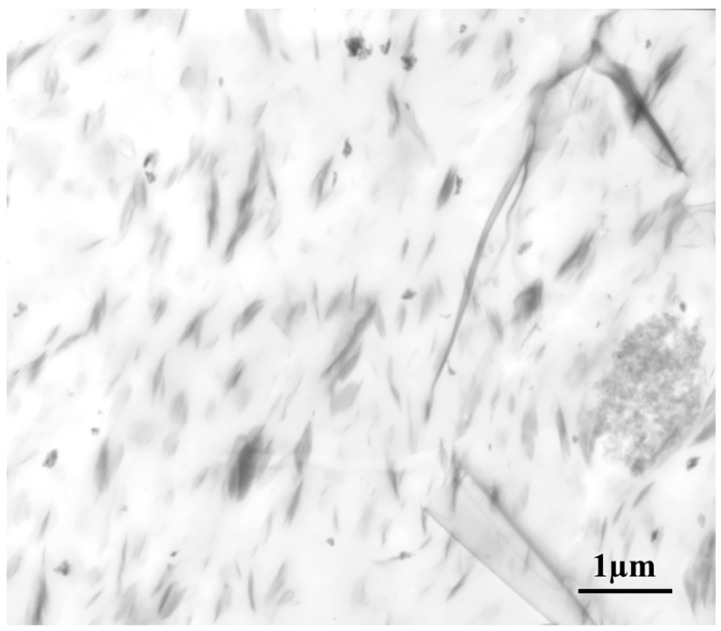
Transmission electron microscopy (TEM) image of the nanorubber highlighting the dispersed and intercalated structure of the layered silicates.

Figure 2 shows a typical TEM image of an EPDM/CR nanorubber with layered silicates. Most of the layered silicates are well dispersed within the EPDM/CR rubber matrix and a large portion of the organic-modified silicate-layer particles appear to be intercalated. The dispersion of the layered silicate was difficult at high silicate loadings; however, the high applied shear force was sufficient to disperse the silicate and allow diffusion of the polymer-matrix molecules between the silicate layers. Both TEM and XRD confirm that layers of organosilicate particles were successfully intercalated with the EPDM/CR-nanorubber molecules.

### 2.2. Mechanical Properties

**Figure 3:**
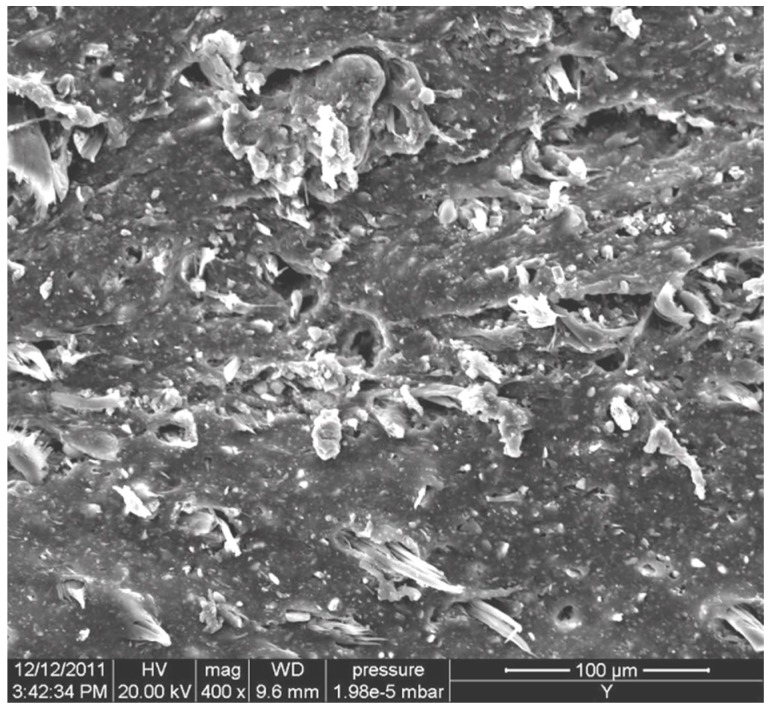
Scanning electron microscopy (SEM) image of a fractured surface of the EPDM/CR nanorubber.

Table 1 lists the hardness and tensile properties of the EPDM/CR nanorubbers; hardness was observed to increase slightly upon addition of the layered silicate. The tensile properties of the EPDM/CR nanorubbers were strongly anisotropic; the tensile strengths in the machine direction (MD) were higher than those in the transverse direction (TD), while elongations in the MD were much lower than those in the TD. As the addition of layered silicates did not influence the tensile strength and elongation, we conclude that Kevlar-fiber orientation is the main factor that affects these properties and that the fibers are highly oriented in the MD. Figure 3 shows an SEM image of a tensile-fractured surface of the EPDM/CR nanorubber; the Kevlar fibers and layered silicates are well dispersed and enclosed by the rubber matrix.

### 2.3. Crosslinking Density

The crosslinking density was determined from the equilibrium swelling [27]. Cured rubber samples were placed in toluene at 25 °C for 48 h. The solvent-uptake percentage, Q and the volume fraction of EPDM/CR in the swollen gel, V_r_, were calculated using Equations (1) and (2), respectively:

Q = (M_sw_ − M_i_)/M_i_, (1)

V_r_ = (1/D_sam_)/[(1/D_sam_) + (Q/D_sol_)], (2)

where M_i_ and M_sw_ are the weights of the rubber sample before immersion in the solvent and in its swollen state, respectively and D_sam_ and D_sol_ are the densities of the rubber sample and solvent, respectively (0.87 g/cm^3^ for toluene). The crosslinking density of the sample, ν, is defined as the number of elastically active chains per unit volume and was calculated by applying the Flory-Rehner equation:

ν = −[ln (1 − V_r_) + V_r_ + χV_r_^2^] / [V_s_ (V_r_^1/3^ − V_r_/2)], (3)

where V_s_ is the molar volume of the swelling solvent (106.1 cm^3^/mol for toluene) and χ is the Flory-Huggins (rubber-toluene) interaction parameter, which was 0.435 for the EPDM/CR-toluene system. Determining the thermodynamic parameters for rubber elasticity is crucial in order to obtain a deeper understanding of mixing in the EPDM/CR/layered-silicate nanorubbers. The expansion of rubber in the presence of a solvent considerably modifies its conformational entropy (ΔS) and elastic Gibbs free energy (ΔG). The elastic Gibbs free energy was determined using the Flory-Huggins equation:

ΔG = RT[ln(1 − V_r_) + V_r_ + χV_r_^2^], (4)

where R is the Boltzmann constant and T is the absolute temperature. From the statistical theory of rubber elasticity, the conformational entropy ΔS is obtained from the equation ΔG = −TΔS, assuming that there are no changes in the internal energy of the network upon stretching.

The crosslinking densities of EPDM/CR nanorubbers with various layered-silicate contents are given in Table 1. The crosslinking density is observed to increase with increasing layered-silicate content, which is attributable to rubber vulcanization, that is, strong chemical and physical bonding between the rubber molecules and the layered silicates, which also explains the observed increase in hardness. Layered silicate is believed to reduce the payne effect of nanorubbers and enhance the mechanical (tensile and hardness) properties. The influence of these interactions on NG migration in the EPDM/CR nanorubbers is discussed below.

### 2.4. Barrier Properties

**Figure 4:**
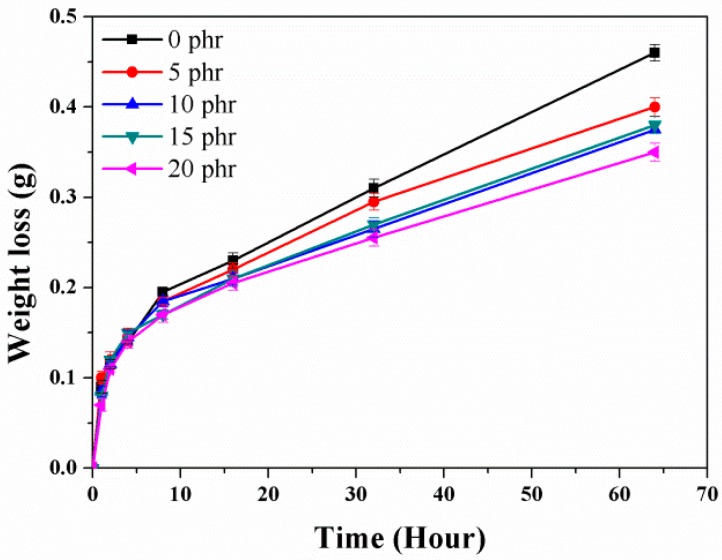
Triacetin-permeation in EPDM/CR nanorubbers with various layered-silicate contents.

Experimental triacetin permeation curves for the nanorubbers at 70 °C are presented in Figure 4. Two regions exhibiting different behavior were observed. In the first region (over the first 4 h), referred to as the “transient state,” nonzero transfer of matter by diffusion occurs and the penetrant concentration is a function of position and time. In the second region (after 8 h), referred to as the “steady state,” constant molecular flux through the membrane occurs and concentration does not vary with time; weight loss decreases with increasing layered-silicate content. The silicate layers act as excluded volumes for the sorption process and are impermeable barriers for diffusion. This is attributed to a more intercalated structure and larger intercalated-space gallery, which lengthen the penetration path for triacetin (TA) vapor. In addition, nanorubbers with higher crosslinking densities are more molecularly compact, which reduces the diffusion of TA vapor within the nanorubbers and the rubber swelling caused by TA. It is evident that reductions in the mobilities of the polymer chains resulting from crosslinking are accompanied by a marked decrease in the ability of TA molecules to diffuse into the polymer; that is, more compact nets inhibit TA migration. The permeability at a given temperature is derived from the slope of the permeation curve in the steady state. The relative permeability is defined as the measured permeability divided by the pure-rubber permeability; values for the various nanorubbers are listed in Table 1. The TA-vapor permeabilities of the nanorubber (20 phr) and pure rubber are 6.80 × 10^−2^ and 10.80 × 10^−2^ g/m·day, respectively; the permeability is lower by 37% (from 100% to 63%) when 20 phr of the layered silicate is included.

**Figure 5:**
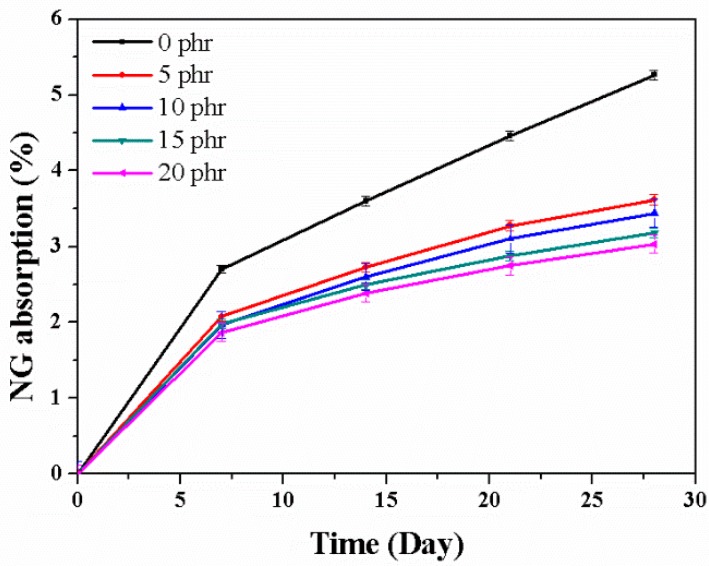
Nitroglycerin adsorption in EPDM/CR nanorubbers with various layered-silicate contents.

Figure 5 shows increases in sample weight as functions of time due to NG migration and adsorption; the observed behavior is similar to that observed for TA permeation, with rapid migration and adsorption rates during the first week; the rates steadily decline after the first week. The weight gain resulting from NG absorption decreased with increasing layered-silicate content; after 28 days, NG absorption had decreased from 5.26% for pure rubber to 3.62% (5 phr), 3.44% (10 phr), 3.19% (15 phr) and 3.03% (20 phr). The addition of layered silicates resulted in a 31%–42% reduction in NG migration, which may be due to the delaminated-platelet structure of the layered silicates that decreases the permeating ability of NG by preventing NG molecules from coming into contact with rubber molecules.

### 2.5. Flammability

**Figure 6:**
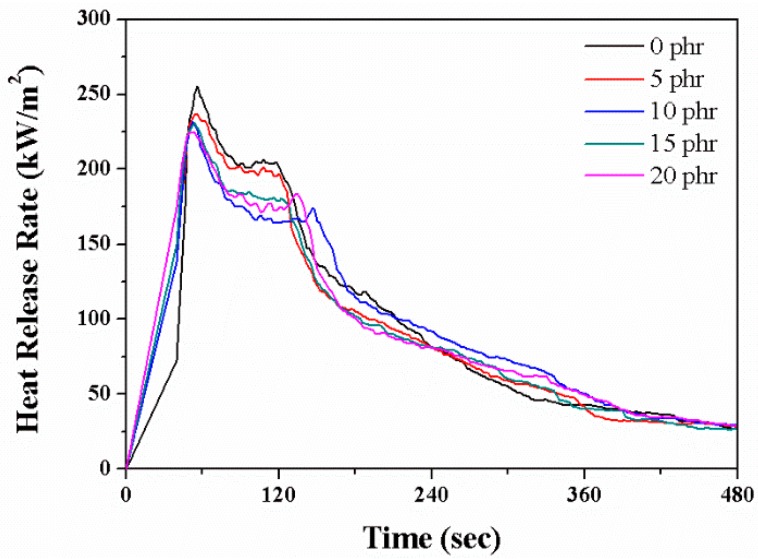
Typical heat release rate (HRR) curves for EPDM/CR nanorubbers with various layered-silicate contents.

**Figure 7:**
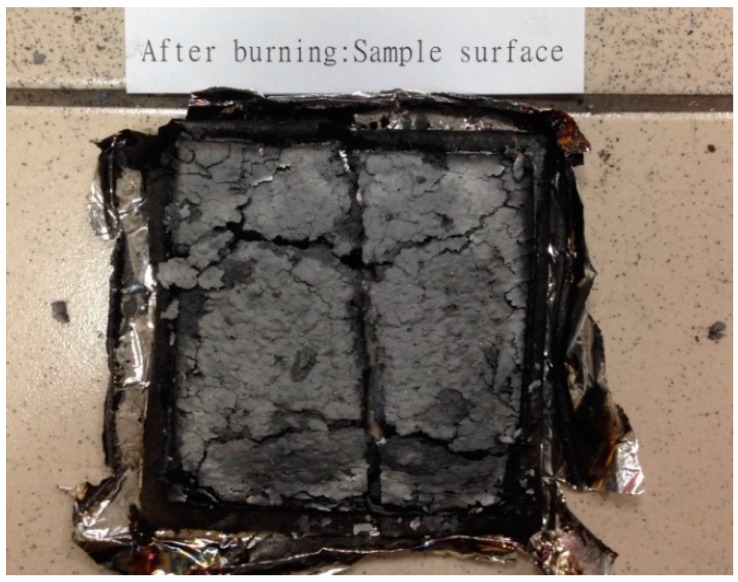
Photographic image showing char formation on an EPDM/CR nanorubber (10 phr layered silicates) after burning.

The flammability of the EPDM/CR nanorubbers were measured using a cone calorimeter. Typical heat-release-rate (HRR) traces for nanorubbers with and without layered silicates are shown in Figure 6 and the peak HRR (pHRR) and average HRR (avgHRR) values are listed in Table 2. pHRR dropped from approximately 255.4 kW/m^2^ to 224.8 kW/m^2^ upon the addition of 20-phr layered silicates, whereas the avgHRR dropped from approximately 181.7 kW/m^2^ to 170.2 kW/m^2^. The formation of a silicate-reinforced carbonaceous char (Figure 7) during combustion, which acts as a barrier to fuel release, lengthens the burn times of the nanorubbers and lowers their HRRs. Longer decomposition-product residence times provide opportunities for other secondary reactions to occur, which may contribute to the formation of thicker char layers. The char structure is essential for lowering flammability and is thought to consist of a multilayered carbonaceous silicate structure that acts as an excellent insulator and mass-transport barrier; this structure contains tortuous paths that not only slow down the escape of volatile products and combustible gases but also limits the amount of oxygen-rich air that enters the sample.

## 3. Materials and Methods

### 3.1. Materials Preparation

EPDM (Royalene 580 HT, Lion Elastomers, Geismar, LA, USA) with a high propylene content and 2.7 wt % ethylidenenorborene (ENB) and CR (Neoprene^®^ GW, DuPont, Hayward, LA, USA) were blended together to form a rubber matrix; a 75:25 ratio of EPDM to CR was used. The contents of the layered silicate (Cloisite^®^ 30B, Southern Clay, Gonzales, TX, USA) were in the 5–20 phr range. The quaternary ammonium salt is used as the modifier with a concentration of 90 mmol/100 g clay. The interlayer distance of the organosiliates is 1.834 nm (2θ = 4.81). Kevlar pulp (1F 1234, DuPont, Hayward, LA, USA) and hydrated silica (Hi-Sil 233, Degussa, Frankfurt, Germany) were used as reinforcing materials. Highly fibrillated Kevlar fibers with 14 μm in diameter, 0.5–1 mm in length and specific surface area of 7–11 m^2^/g were used in this work. The fibrillated Kevlar fibers were pre-mixed within CR rubbers to assure well disperse and good interfacial bonding to rubber matrix. Kevlar pulp agglomerates were shattered and Kevlar short fibers were dispersed into rubber matrix uniformly due to the intense shear force result from the Banbury mixer and two-mill roller. To obtain the required physical and chemical properties, the formulation also contained other additives, including ablating agents, antioxidants, processing aids, activators and curing agents, all of which were supplied from a local agency (R.T. Vanderbilt Co. Ltd., Taiwan). The composition used in this study is given in Table 3.

### 3.2. The Blending Process

EPDM and CR were blended in a Banbury mixer at 150 °C for 30 s, after which the reinforcing and ablating agents and the other additives (except the curing agents) were added and mixed for 4 min. The compounds were subsequently sheeted using a two-mill roller. After aging for 3 h, the curing agents were added and dispersed using a two-mill roller to form 2-mm-thick sheets. Because Kevlar fibers were added, the properties of the nanorubber will be strongly anisotropic. Therefore, samples were marked and prepared in the machine (MD) and transverse (TD) directions.

### 3.3. Sample Preparation and Characterization

After aging at room temperature for 24 h, the samples were cured in a hot press at 160 °C at a pressure of 150 kg/cm^2^ for various curing times, t_90_, which were derived from a Monsanto oscillating disc rheometer (MDR 2000). Then, 2-mm-thick nanorubber sheets were subsequently prepared in both the MD and TD directions. Tensile testing was performed using a material testing machine (Sintech, MTS, Eden Prairie, MN, USA) at a crosshead speed of 50 cm/min, according to ASTM D 412. Five specimens were tested for each data point and average values were obtained. Hardness measurements were conducted according to ASTM D2240 using a durometer hardness tester (Tecolok Co. Inc., Tokyo, Japan)

X-ray diffraction (XRD) measurements were carried out on a D5000 diffractometer (Siemens, Munich, Germany) with Ni-filtered Cu K_α_ radiation (λ = 0.1542 nm). The samples were scanned in step mode at a scan rate of 1.5°/min at 2θ < 10°. Scanning electron microscopy (SEM, JSM-6390, JEOL, Tokyo, Japan) images were obtained at an accelerator voltage of 20 kV. A layer of gold was deposited on the surface of each cured nanorubber sample using a JEOL JFC-1200 fine-coater. Transmission electron microscopy (TEM) was performed on a JEOL JEM 2000FX electron microscope operating at an accelerator voltage of 120 kV. Ultrathin sections of the cured samples were microtomed into ~100 nm slices using a Leica Ultracut UCT and a diamond knife prior to TEM experiments. A layer of carbon was deposited onto these slices that were then placed on a 400-mesh copper net.

Because of the high explosion risk associated with using NG, triacetin (TA), which has a similar chemical structure to NG, was used as the migrating agent. The TA-permeability properties for the EPDM/CR nanorubbers were evaluated according to ASTM E96; this method determines the amount of liquid crossing a material by the weight loss of a metallic cell that contains the liquid and is surrounded by the polymer membrane to be studied. The effects of layered silicates on the TA-vapor permeability of the EPDM/CR nanorubbers were determined from the TA weight loss. EPDM/CR-nanorubber specimens with diameters of 46 mm and thicknesses of 2 mm were prepared and used to seal a 200 mL beaker filled with 100 mL of TA. The assembly was placed in an oven at 70 °C and the amount of evaporated TA was determined by measuring the overall weight loss with a precision balance.

**Figure 8:**
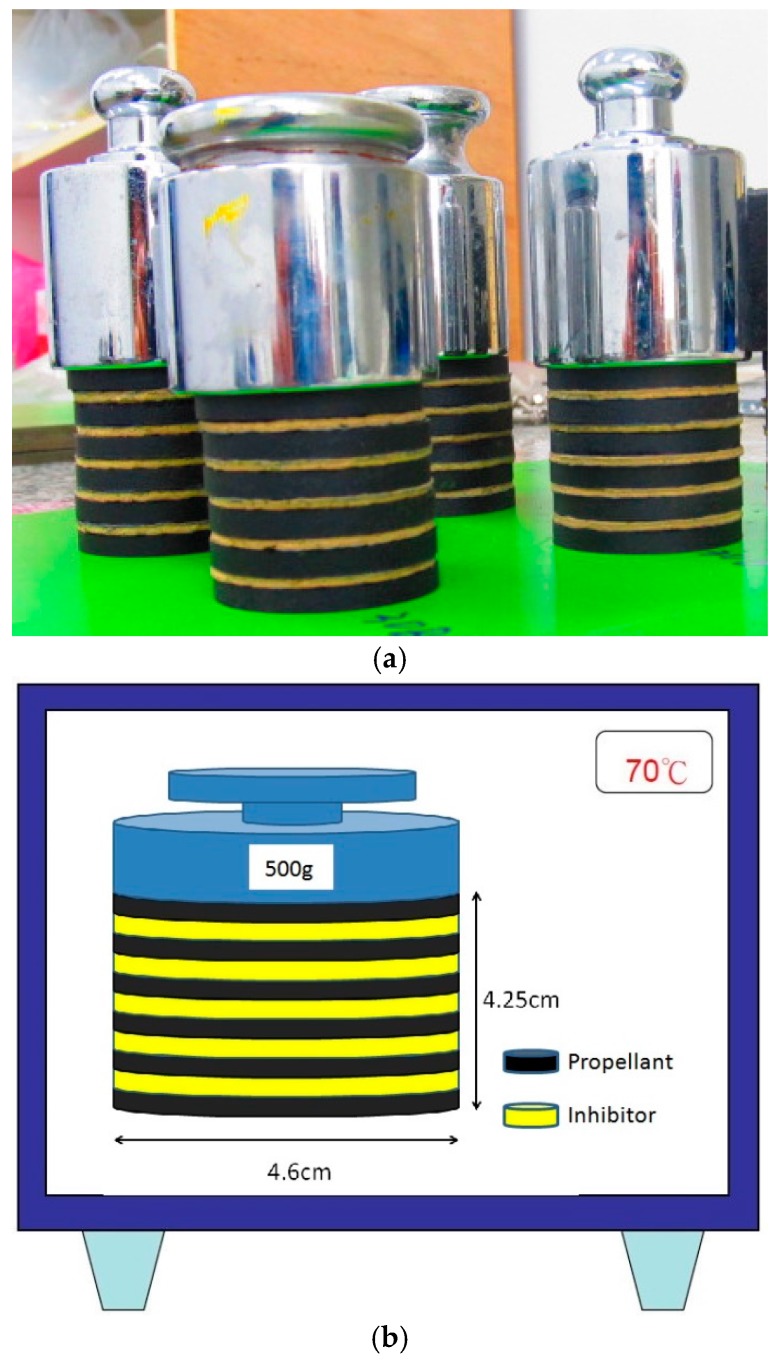
(**a**) Images of test specimens used in the nitroglycerine (NG)-migration and absorption experiments; (**b**) Illustration depicting the experimental set up for NG-migration and absorption experiments.

The NG-barrier properties of the EPDM/CR nanorubbers were evaluated by measuring the NG absorptions of the nanorubbers [28,29,30,31,32,33,34]. As shown in Figure 8a, piled specimens, composed of six layers of propellant (in black) and five layers of nanorubber (in yellow), were prepared. The diameter of each sample was 46 mm and the nanorubber and propellant layers were 2.5-mm and 5-mm thick, respectively. A 500 g counterweight was placed on the top of the specimen, which was then placed in an oven at 70 °C, as shown in Figure 8b. The weight change of each nanorubber layer was measured daily at room temperature. The NG-absorption uptake percentage, A, is defined as:

A = (M_f_ − M_o_)/M_o_, (5)

where M_o_ refers to the weight of the rubber sample before contact with the propellant and M_f_ refers to the weight of the rubber sample after contact with the propellant at 70 °C. The experiment was terminated when the interface between the propellant and nanorubber became tacky or when fracturing was observed in the nanorubber layer.

A cone calorimeter enables the flammability of a material to be quantitatively analyzed [35]. Cone specimens were 2-mm-thick, 10 cm × 10 cm squares. The materials were tested at a heat flux of 100 kW/m^2^. The specimens were placed in an aluminum foil pan with a lip 13 mm higher than the top of the sample surface. Average heat-release rate (avgHRR) and peak heat-release rate (pHRR) were used to evaluate flame retardancy.

## 4. Conclusions

The barrier properties of layered-organosilicate reinforced EPDM/CR nanorubbers, in terms of their triacetin and nitroglycerin permeabilities, were investigated the first time. EPDM/CR/layered-silicate nanorubbers with intercalated structures were prepared by the melt-compounding method. Tensile properties were dominated by the addition and orientation of Kevlar fibers but were less affected by the layered silicates. Triacetin-vapor permeability decreased with increasing layered-silicate content. The penetration path for TA vapor lengthened due to the intercalated layered silicates and higher molecular compaction (caused by higher crosslinking density). The relative TA-vapor permeability of the 20-phr nanorubber was reduced by 37% compared to pure rubber. The weight gain resulting from nitroglycerin absorption decreased with increasing layered-silicate content, which highlights the barrier effect of the layered silicate within the nanorubber. The layered silicates also improved the flame retardancies of the nanorubbers through the formation of silicate-reinforced carbonaceous chars. Hence, layered-silicate reinforced EPDM/CR nanorubbers are potentially good rocket-propellant inhibitors.

## Figures and Tables

**Table 1:** Mechanical properties, crosslinking densities and triacetin (TA) permeabilities of layered-silicate reinforced EPDM/CR nanorubbers.

Layered silicate (phr)	0	5	10	15	20
Hardness	87	90	90	88	91
Tensile strength (MD), MPa	10.3 ± 0.2	11.7 ± 0.5	11.0 ± 0.6	11.7 ± 0.5	11.7 ± 0.4
Elongation (MD), %	31.1 ± 2.4	24.1 ± 2.8	25.1 ± 4.9	26.7 ± 2.4	21.0 ± 2.6
Tensile strength (TD), MPa	6.9 ± 0.1	7.9 ± 0.4	10.2 ± 0.6	7.0 ± 0.2	8.7 ± 0.2
Elongation (TD), %	60.5 ± 22.2	50.5 ± 21.2	47.2 ± 4.4	54.0 ± 14.8	52.4 ± 4.3
Crosslinking density × 10^−2^ (mol/cm^3^)	3.35	3.41	3.49	3.56	3.70
Relative permeability	1	0.77	0.72	0.75	0.63

**Table 2:** Flammability properties of layered-silicate reinforced EPDM/CR nanorubbers.

**Layered Silicate(phr)**	**avgHRR (kW/m^2^)**	**pHRR (kW/m^2^)**
0	181.7	255.4
5	174.7	236.9
10	171.0	232.2
15	168.2	231.4
20	170.2	224.8

**Table 3:** EPDM/CR formulation.

Function	Ingredient	Composition in Phr
Rubber	EPDM Neoprene	75 25
Filler	Organosilicate Barium sulfate Magnesium oxide Diatomaceous Silica	0–20 (various) 7.5 3 15 20
Flammability resistant materials	Kevlar fibers Decabromethylether Antimony trioxide	10 5 7.5
Processing aids	Zinc oxide Stearic acid Wax dispersant Phosphate ester silane Dispersant	5 1 1 4 1.5 1.5
Antioxidants	Antioxidant, RD Naguard, 445 Antioxidant, RQ01 Microwax	1.5 1.5 3 0.5
Curing agents	Peroxide, F40KE Cure Co-agent, SR-350 Accelerator	6 2 2 0.2
